# VEGF-C mRNA和EGFR mRNA在非小细胞肺癌肿瘤组织和淋巴结组织中的表达及临床意义

**DOI:** 10.3779/j.issn.1009-3419.2010.12.09

**Published:** 2010-12-20

**Authors:** 燕 管, 其森 郭, 洪生 曾

**Affiliations:** 250117 济南，山东省医学科学院，山东省肿瘤医院内科 Department of Internal Medicine, Shandong Tumor Hospital, Jinan 250117, China

**Keywords:** 肺肿瘤, 血管内皮生长因子C, 表皮生长因子受体, 病理特征, Lung neoplasms, Vascular endothelial growth factor-C, Epidermal growth factor receptor, Pathologic characteristic

## Abstract

**背景与目的:**

已有的研究表明一些分子标志物在肺癌的诊断、预测疗效、判断预后中起着重要作用。本研究旨在探讨血管内皮生长因子C（vascular endothelial growth factor-C, VEGF-C）mRNA、表皮生长因子受体(epidermal growth factor receptor, EGFR）mRNA在非小细胞肺癌（non-small cell lung cancer, NSCLC）患者肿瘤组织和淋巴结组织中的表达及临床意义。

**方法:**

采用RT-PCR与荧光定量PCR技术检测VEGF-C mRNA、EGFR mRNA在NSCLC患者肿瘤组织和淋巴结组织中的表达情况，并分析肿瘤组织及淋巴结组织中两因子的表达与临床病理特点的联系及它们之间的相关性。

**结果:**

VEGF-C mRNA和EGFR mRNA在肺癌组患者肿瘤组织及淋巴结组织中表达水平高于良性肺病对照组（*P* < 0.05）；淋巴结转移组表达水平明显高于无淋巴结转移组（*P* < 0.05）；VEGF-C、EGFR在肿瘤组织中与淋巴结组织中mRNA的表达明显相关（*r*=0.834, *P* < 0.001; *r*=0.817, *P* < 0.001）。

**结论:**

*VEGF-C*、*EGFR*基因高表达与淋巴结转移有关，有助于肺癌的早期诊断、预测疗效及预后判断。

## Abstract (en)

**Background and objective:**

It has been proven that some molecular markers play important roles in diagnosis, clinical prediction and prognosis monitoring for lung cancer. The aim of this study is to investigate the expressions and their significances of vascular endothelial growth factor-C (VEGF-C) mRNA and epidermal growth factor receptor (EGFR) mRNA in non-small cell lung cancer (NSCLC) tissues and lymph node tissues.

**Methods:**

The expressions of VEGF-C mRNA and EGFR mRNA were detected in tumor tissues and lymph node tissues by reverse transcription polymerase chain reaction (RT-PCR) and real-time fluorescence quantitative PCR. The relationship and correlation between the expressions of VEGF-C mRNA, EGFR mRNA and clinicopathologic characteristics were analyzed.

**Results:**

The expressions of VEGF-C mRNA and EGFR mRNA in tumor tissues and lymph node tissues were significantly higher than those in control group (*P* < 0.05). The expressions of VEGF-C mRNA and EGFR mRNA were much higher in patients with lymph node metastasis than those without lymph node metastasis (*P* < 0.05). The expressions of VEGF-C mRNA and EGFR mRNA in tumor tissues and lymph node tissues were significantly correlated (*r*=0.834, *P* < 0.001; *r*=0.817, *P* < 0.001).

**Conclusion:**

The overexpressions of *VEGF-C* and *EGFR* genes are closely related to lymph node metastasis. VEGF-C mRNA and EGFR mRNA are valuable biomarkers for early diagnosis, clinical prediction and prognosis for lung cancer.

肺癌已成为我国恶性肿瘤死亡率之首，其中占肺癌80%的非小细胞肺癌（non-small cell lung cancer, NSCLC）是导致肺癌高发病率和高死亡率的主要原因。血管内皮生长因子C（vascular endothelial growth factor-C, VEGF-C）是一种针对淋巴管内皮细胞的有丝分裂原，具有刺激血管和淋巴管生成的双重作用，其表达与肿瘤的血管生成、临床病理特征和不良预后有关^[1]^。表皮生长因子受体(epidermal growth factor receptor, EGFR）是原癌基因*CerbB-1*的表达产物，与肿瘤的发生、发展密切相关。近年来对EGFR与肿瘤的血管生成、高侵袭性及转移关系的研究越来越多，很多研究^[2, 3]^提示EGFR的高表达往往反映肿瘤的高侵袭力、高转移性及预后不良。本实验利用RT-PCR与荧光定量PCR技术检测NSCLC患者和良性肺病患者的肿瘤组织及淋巴结组织中VEGF-C mRNA和EGFR mRNA的表达水平并分析其相关性，以探讨两因子表达水平与临床病理因素之间的相关性及其与淋巴结转移的关系。

## 1 材料与方法

### 1.1 试剂与仪器

总RNA抽提试剂盒购自Invitrogen公司，反转录试剂盒购自TaKaRa公司，电泳系统购自BIO-RAD公司，荧光定量基因扩增仪7000型购自美国ABI公司。逆转录引物由上海生工生物工程技术服务有限公司合成，*VEGF-C*基因：Forward primer：5'-TCAAGGACAGAAGAGA- CTATAAAATTTGC-3'；Reverse primer：5'-ACTC- CAAACTCCTTCCCCACAT-3'。*EGFR*基因：Forward primer：5'-GGACTCTGGATCCCAGA AGGTG-3'；Reverse primer：5'-GCTGGCCATCACGTAGGCTT-3'。*GAPDH*基因：Forward primer：5'- CA ACAGCCTCA AGATCATCAGC-3'；Reverse primer：5'- TTCTAGACGGCAGGTCAGGTC-3'。

### 1.2 标本来源

选取2008年1月-2009年10月在山东省肿瘤医院胸外科行手术切除的NSCLC患者56例为病例组，良性肺病患者10例为对照组。病例组患者平均年龄59.98岁（36岁-37岁），腺癌26例，鳞癌24例，其它6例，高分化9例，中分化28例，低分化19例，有淋巴结转移38例，无淋巴结转移18例，Ⅰ期13例，Ⅱ期17例，Ⅲ期23例，Ⅳ期3例。对照组患者平均年龄52.61岁（34岁-67岁），肺错构瘤2例，肺炎性假瘤4例，肺结核4例。病例组采集术中切除的新鲜肿瘤组织和淋巴结组织，对照组采集病灶组织。手术结束编号标记后立即冻存于-80 ℃冰箱中备用。

### 1.3 方法

采用TRIzol法从组织中提取总RNA，紫外分光光度仪检测RNA质量和纯度；逆转录总RNA得到cDNA，以cDNA为模板进行PCR扩增出目的片段VEGF-C、EGFR和GAPDH；使用Geneamp Pcr System 9600型PCR扩增仪进行PCR反应，用TAE配制1.5%的琼脂糖凝胶，在110 V电压下扩增40 min，进行PCR产物电泳鉴定；用标准品DNA对PCR产物进行定量，取2 μL PCR产物加入18 μL ddH_2_O，取2 μL稀释后的PCR产物加入18 μL SYBR，引物各0.5 μL，cDNA 2.5 μL，用不含RNA酶的水补至25 μL，短暂离心后进行扩增。扩增条件为95 ℃、10 s，然后以95 ℃ 5 s，57 ℃ 20 s，72 ℃ 30 s循环40次，60 ℃、15 min后检测信号；根据分子量计算出将1 μL样品浓度调整为1×10^9^个拷贝/mL所需加入ddH_2_O的量；分别加入相应体积液体将样品稀释成1×10^9^个拷贝/mL，梯度稀释制作标准曲线，根据样品的CT值可以从标准曲线上读出各个样品的浓度（拷贝数/mL），管家基因的表达相对恒定，计算出目的基因的拷贝数与相同样本的管家基因的拷贝数的比值作为目的基因的表达水平，从而得到计量资料。

### 1.4 统计学处理

应用SPSS 15.0软件进行数据的统计分析。所有计量资料均应先经过单样本*K-S*检验，判断是否为正态分布。符合双样本均为正态分布的计量资料比较采用*t*检验、直线相关分析等方法，*P* < 0.05为差异有统计学意义。

## 2 结果

### 2.1 RT-PCR检测VEGF-C和EGFR在肿瘤组织和淋巴结组织中的表达情况

以cDNA为模板，分别以VEGF-C、EGFR和G A PDH引物行P CR扩增，扩增出目的片段VEGF-C、EGFR和GAPDH，1.5%的琼脂糖凝胶电泳结果见图 1。

**1:**
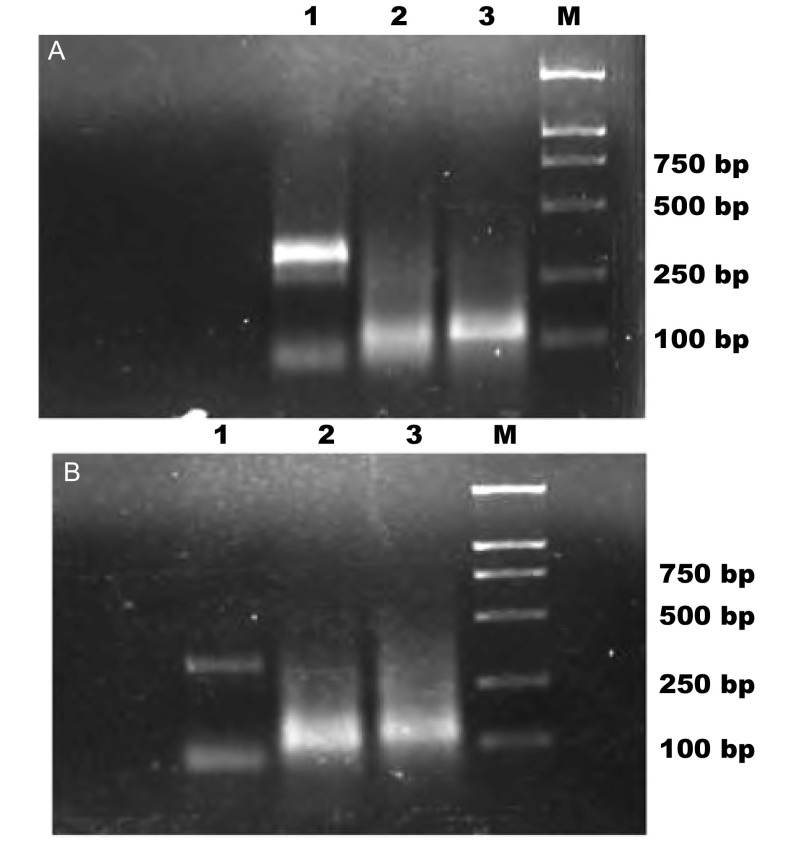
VEGF-C和EGFR在肿瘤组织（A）和淋巴结组织（B）中的表达。1：GAPDH；2：VEGF-C；3：EGFR；4：DNA marker。 Expressions of VEGF-C (A) and EGFR (B) in tumor tissues and lymph node tissues. 1: GAPDH; 2: VEGF-C; 3: EGFR; 4: DNA marker.

### 2.2 荧光定量PCR检测结果

目的基因*VEGF-C*、*EGFR*与管家基因的溶解曲线（图 2）的定量结果显示，VEGF-C mRNA在肺癌组患者肿瘤组织及淋巴结组织中的表达水平高于良性肺病组（59.6±12.5 *vs* 42.8±8.5, *P* < 0.05），淋巴结转移组表达水平高于无淋巴结转移组（62.3±15.3 *vs* 48.2±12.6, *P*=0.001），肺癌组织中与淋巴结组织中VEGF-C mRNA表达水平呈同向变化，直线相关分析发现VEGF-C在肿瘤组织中的表达水平与淋巴结组织中的表达水平明显相关（*r*=0.834, *P* < 0.001）（图 3A）。EGFR mRNA在肺癌组患者肿瘤组织中的表达水平高于良性肺病组（6.27±0.96 *vs* 5.37±0.48, *P*=0.015），淋巴结转移组表达水平高于无淋巴结转移组（6.19±0.90 *vs* 5.15±0.86, *P*=0.012），直线相关分析发现EGFR在肿瘤组织中的表达水平与淋巴结组织中的表达水平明显相关（*r*=0.817, *P* < 0.001）（图 3B）。

**2:**
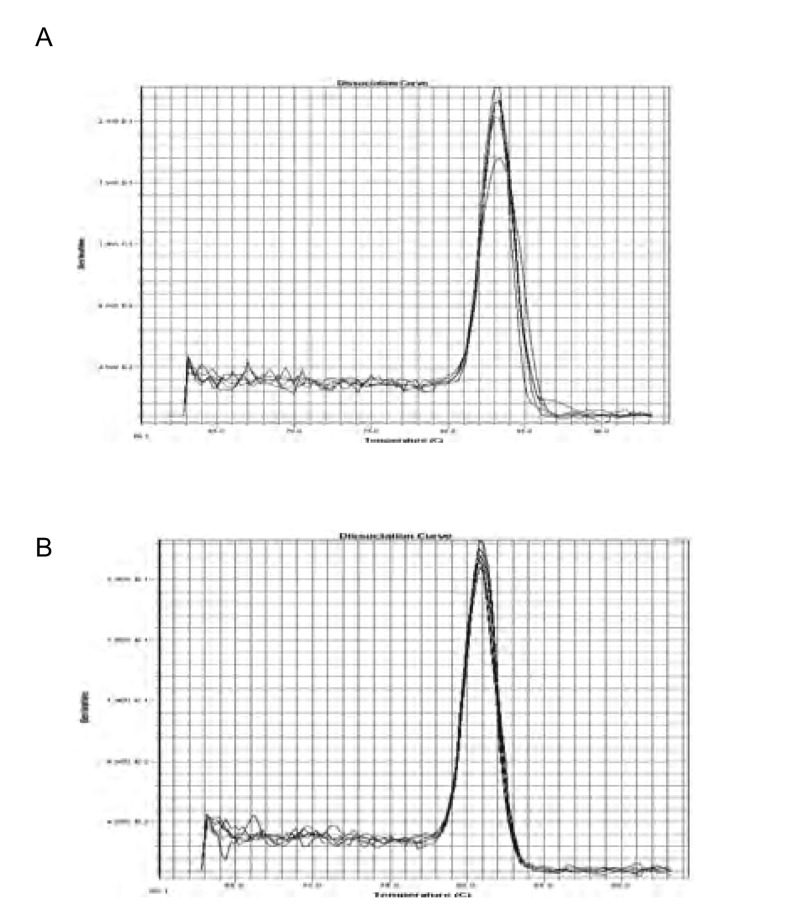
VEGF-C（A）和EGFR（B）的溶解曲线 Dissolving curve of VEGF-C (A) and EGFR (B)

**3:**
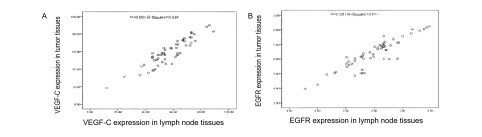
VEGF-C（A）和EGFR（B）在肿瘤组织与淋巴结组织中表达的相关性 VEGF-C (A) and EGFR (B) expressions in tumor tissues and lymph node tissues

## 3 讨论

肿瘤的转移、复发是一个多因子参与的复杂过程，新生血管和淋巴管的生成在肿瘤的发生和转移过程中起着重要的作用。一些分子标志物的检测可以指导治疗、判断预后，进而使患者获益更大。VEGF家族是高度特异的内皮细胞有丝分裂原，可特异性促进内皮细胞生长及血管生成，增加血管的通透性，有助于肿瘤侵袭转移，是目前诱导肿瘤血管生成作用最强、特异性最高的血管生长因子^[4]^。大多数肿瘤细胞本身、肿瘤浸润的巨噬细胞和肥大细胞能分泌高水平VEGF。VEGF-C由7个外显子组成，是一种针对淋巴管内皮细胞的有丝分裂原，具有刺激血管和淋巴管生成的双重作用。多项研究提示，人类肿瘤表达的VEGF-C与肿瘤的转移播散相关，且肿瘤进展过程与淋巴管生成有关，淋巴管生成已成为目前肿瘤转移研究的热点。Zhang等^[5]^应用实时定量聚合酶链反应技术研究VEGF-C在人乳腺癌组织中的表达，结果显示VEGF-C mRNA表达与肿瘤淋巴结转移明显相关，并认为VEGF-C表达是临床评价乳腺癌淋巴侵袭扩散的指标之一。Mylona^[6]^研究了乳腺癌中VEGF-C蛋白表达与淋巴管、血管侵犯与肿瘤增殖的关系，结果显示VEGF-C的表达更多的是通过脉管容量的增加而不单是通过淋巴管数量的增加来发挥作用。Li^[7]^利用RT-PCR、免疫组化方法检测了VEGF-C在52例NSCLC患者手术切取的肿瘤组织和淋巴结组织中的表达，结果表明VEGF-C mRNA、VEGF-C蛋白表达水平与淋巴结转移相关，可作为预测NSCLC淋巴结转移的相关指标。

本研究选择新鲜的冰冻肿瘤组织和淋巴结组织进行VEGF-C和EGFR表达研究，新鲜冰冻组织与石蜡包埋的组织相比为更好的研究对象，研究结果更可靠^[8]^。在实时荧光定量PCR反应中，本研究引入了一种荧光化学物质，随着PCR反应的进行，PCR反应产物不断累积，荧光信号强度也等比例增加。每经过一个循环，收集一个荧光信号强度，这样我们就能通过荧光信号强度的变化监测产物量的变化。表示每个PCR反应管内荧光信号到达设定的阈值时所经历的循环数即为CT值。我们通过获得未知样品的CT值，然后从标准曲线上读出样品的拷贝数。本研究分析了VEGF-C与临床病理特点的联系，表明VEGF-C无论是在肿瘤组织中还是淋巴结组织中的表达均与淋巴结转移有关，VEGF-C可以作为NSCLC淋巴结转移的预测指标。肿瘤细胞内VEGF-C的过表达促进了肿瘤内淋巴管的生成，从而促进区域淋巴结的转移。我们对肿瘤组织和淋巴结组织中VEGF-C表达的分析表明：VEGF-C mRNA的表达在有淋巴结转移组和无淋巴结转移组有统计学差异（*P*=0.001）。NSCLC患者与良性肺部疾病患者肿瘤组织中与淋巴结组织中EGFR mRNA的表达均有统计学差异（*P*=0.001）。VEGF-C无论是在肿瘤组织中还是淋巴结组织中的表达，均与NSCLC的淋巴结转移存在相关性。目前癌细胞经淋巴道播散的机制尚不明了，但VEGF-C与淋巴管形成的相关性是明确的，其导致转移增高的原因可能包括：①VEGF-C与淋巴管内皮细胞的VEGFR-3结合，诱导VEGFR-3酪氨酸激酶磷酸化，增加瘤内、瘤周淋巴管的数目和管径，提升了肿瘤淋巴转移机会^[9]^；②VEGF-C可使血管、淋巴管的渗透性增加，可能是通过VEGFR-2和VEGFR-3介导实现的^[10]^；③VEGF-C通过改变肿瘤细胞与胞外基质的粘附性，提高癌细胞的粘附作用；④VEGF-C可作为一种趋化因子，这可能与其特殊的蛋白溶解酶作用有关，一方面增强肿瘤和特异器官的亲和性，另一方面促进癌细胞向淋巴管方向移动，实现转移。总之，VEGF-C参与人NSCLC的浸润、转移，但关于VEGF-C在肺癌发生、发展中的机制及与预后的关系尚需进一步研究。

EGFR是一种具有酪氨酸激酶活性的膜表面传感器，普遍表达于人体的表皮细胞和基质细胞，并在多种人类恶性肿瘤中高表达，其所介导的信号转导效应具有多向性，包括增殖、迁移、细胞分化和内环境的稳定等，与细胞的再生和恶性肿瘤的发生、发展密切相关。Pore等^[11]^研究表明，在晚期浆液性低分化卵巢癌中EGFR过度表达和肿瘤血管发生有关，肿瘤细胞有血液供应增殖更加迅速。Fresno等^[12]^使用RT-PCR技术检测结果显示EGFR在NSCLC组织中高表达，EGFR在NSCLC组织中的表达率明显高于良性肿瘤和正常组织（*P* < 0.05），表明EGFR在肺肿瘤发生过程中可能起到了一定的作用。Tomov等^[13]^采用免疫组化方法研究了EGFR在良、恶性卵巢肿瘤中的表达情况，结果发现恶性卵巢组织EGFR的表达率明显高于良性组织，差别有统计学意义；有腹水和进展期患者的EGFR表达率更高，研究表明卵巢癌细胞中EGFR的过度表达导致侵袭力增加。

本研究结果发现肿瘤组织及淋巴结组织中EGFR mRNA的表达在淋巴结转移组和无淋巴结转移组有统计学差异，是EGF R表达与肺癌淋巴结转移有关的直接证据，表明EGFR参与了NSCLC的发展过程，所以，EGFR高表达往往反映了肿瘤的高侵袭力、高转移性及预后不良，EGFR可以作为肺癌早期诊断转移或微转移，判断预后的预测指标。本研究还表明NSCLC患者与良性肺部疾病患者的肿瘤组织与淋巴结组织中EGFR mRNA的表达水平均有统计学差异（*P*=0.015, *P*=0.020），但是目前关于肺癌患者与良性肺病两组之间差异的报道还存在一定的矛盾^[14]^。

本研究中肿瘤组织和淋巴结组织中VEGF-C mRNA的表达经直线相关分析具有相关性，且肿瘤组织和淋巴结组织中EGFR mRNA的表达经直线相关分析亦具有相关性，提示VEGF-C和EGFR可能具有相似的生物学标记的意义，这将为采用淋巴结组织替代肿瘤组织检测VEGF-C和EGFR提供了理论依据，有可能为患者提供一种更快捷、简便的替代检测方法，有助于减轻患者痛苦和经济负担，并对肺癌的早期诊断转移或微转移、评价预后及多靶点个体化治疗的选择奠定了理论基础。
